# *In vitro*, *in vivo* and *ex vivo* demonstration of the antitumoral role of hypocretin-1/orexin-A and almorexant in pancreatic ductal adenocarcinoma

**DOI:** 10.18632/oncotarget.24084

**Published:** 2018-01-09

**Authors:** Stéphanie Dayot, Daniela Speisky, Anne Couvelard, Pierre Bourgoin, Valérie Gratio, Jérôme Cros, Vinciane Rebours, Alain Sauvanet, Pierre Bedossa, Valérie Paradis, Philippe Ruszniewski, Alain Couvineau, Thierry Voisin

**Affiliations:** ^1^ INSERM UMR1149 Centre de Recherche sur l’Inflammation (CRI), Université Paris-Diderot, Sorbonne Paris Cité, DHU UNITY, Faculté de Médecine Xavier Bichat, Huchard, 75018 Paris, France; ^2^ Département de Pathologie Beaujon-Bichat, AP-HP, Hôpital Bichat, Huchard, 75018 Paris, France; ^3^ Service de Pancréatologie-Gastroentérologie PMAD, Pôle des Maladies de l’Appareil Digestif, AP-HP, Hôpital Beaujon, 92118 Clichy, France; ^4^ Département de Pathologie Beaujon-Bichat, AP-HP, Hôpital Beaujon, 92118 Clichy, France

**Keywords:** pancreatic cancer, orexin receptor, GPCR, apoptosis, patient-derived xenograft

## Abstract

Pancreatic ductal adenocarcinoma (PDAC) is still the poorest prognostic tumor of the digestive system. We investigated the antitumoral role of orexin-A and almorexant in PDAC. We analyzed the orexin receptor type 1 (OX1R) expression by immunohistochemistry in human normal pancreas, PDAC and its precursor dysplastic intraepithelial lesions. We used PDAC-derived cell lines and fresh tissue slices to study the apoptotic role of hypocretin-1/orexin-A and almorexant *in vitro* and *ex vivo*. We analyzed *in vivo* the hypocretin-1/orexin-A and almorexant effect on tumor growth in mice xenografted with PDAC cell lines expressing, or not, OX1R. Ninety-six percent of PDAC expressed OX1R, while adjacent normal exocrine pancreas did not. OX1R was expressed in pre-cancerous lesions. *In vitro*, under hypocretin-1/orexin-A and almorexant, the OX1R-positive AsPC-1 cells underwent apoptosis, abolished by the tyrosine phosphatase SHP2 inhibitor, NSC-87877, whereas the OX1R-negative HPAF-II cell line did not. These effects were mediated by phosphorylation of OX1R and recruitment of SHP2. *Ex vivo*, caspase-3 positive tumor cells were significantly higher in fresh tumour slices treated 48h with hypocretin-1/orexin-A, as compared to control, whereas cellular proliferation, assessed by Ki-67 index, was not modified. *In vivo*, when AsPC-1 cells or patient-derived cells were xenografted in nude mice, hypocretin-1/orexin-A or almorexant, administrated both starting the day of cell line inoculation or after tumoral development, strongly slowed tumor growth. Hypocretin-1/orexin-A and almorexant induce, through OX1R, the inhibition of PDAC cellular growth by apoptosis. Hypocretins/orexins and almorexant might be powerful candidates for the treatment of PDAC.

## INTRODUCTION

Pancreatic ductal adenocarcinoma (PDAC) is the eleventh most frequent cancer in the United States, accounting for 53,070 new cases per year, and it will stand as the second cause of cancer related-death by 2030 [1–4]. The low 5-year survival rate (about 7%) is due to highly invasive behavior with frequent non-resectability at initial diagnosis. Moreover this tumor is mainly drug-resistant to classic chemotherapeutic agents because of the association of a fibrotic, immunosuppressive, and hypoxic microenvironment with numerous tumor mutations that activate proto-oncogene expression, block tumor suppressor genes or interfere with apoptosis [5]. Thus far and despite intense efforts, optimal chemotherapeutic strategies have only slightly improved clinical outcome in patients with PDAC.

G-protein coupled receptors (GPCRs) belong to a large superfamily of cell surface signaling proteins involved in many pathophysiological processes [6]. Diverse GPCRs are overexpressed in tumor cells, and are involved in the initiation and/or progression of cancer by stimulating or inhibiting proliferation and/or apoptosis [7]. We have recently demonstrated the aberrant expression of the orexin receptor sub-type 1, OX1R, in primary colorectal tumors, whereas no expression could be found in normal colon [8].

Orexin-A and orexin-B [9], are two hypothalamic neuropeptides involved in the sleep/wake cycle and feeding behavior [10]. In this context, pharmaceutical industries have developed several orexin antagonists (Dual Orexin Receptors Antagonist or DORA) including almorexant and suvorexant in order to promote the sleep control. Recently, the U.S. Food & Drug Administration (FDA) approved the use of a reversible dual orexin receptor antagonist suvorexant, for the treatment of insomnia. Biological functions of orexins have been described in various peripheral tissues, but remain questionable [11–14]. The effects of orexins are mediated by two GPCR sub-types, OX1R and OX2R [9]. The activation of OX1R and OX2R by orexins led to the intracellular Ca^2+^ mobilization which was inhibited by orexin antagonists. In OX1R expressing cell lines, we have shown that OX1R triggers a robust mitochondrial apoptosis by an original mechanism [8, 15]. Activation of OX1R induces the tyrosine phosphorylation of 2 tyrosine-based motifs, ITIM (immunoreceptor tyrosine-based inhibition motif), of OX1R, which allow the recruitment of the phosphotyrosine phosphatase SHP-2 and the caspase-3 activation [16–18].

PDAC cells gain protection against the apoptotic mitochondrial pathway by overexpressing Bcl-family proteins (Bfl1, BCL-X_L_, MCL-1) [19] or by blocking activation of caspases – e.g., by overexpressing caspase inhibitors (cIAP, XIAP1, survivin), epigenetic downregulation of pro-caspase gene expression or direct caspase inhibition by cysteine nitrosylation [20, 21]. Novel strategies aim at the commitment of PDAC cells to undergo apoptosis efficiently in response to novel drugs. In this context, studying the role and expression of the proapoptotic orexin receptor OX1R in PDAC represents a promising approach. We showed that the receptor OX1R is not present in normal exocrine pancreas but is aberrantly expressed in almost all primary PDAC tested. We also investigated the ability of orexin-A to promote apoptosis: 1) *in vitro* studies by using PDAC cell lines as AsPC-1 cells; 2) *ex vivo* studies by using tissue culture derived from patients. We showed that orexin-A strongly reduced the development of tumors and reversed the growth of developed tumors in nude mice xenografted with human pancreas cancer cell line or patient-derived cells. Surprisingly, almorexant which is a Ca^2+^ pathway DORA antagonist, behaves as full apoptotic pathway agonist. These data indicate that OX1R, OxA and almorexant might be considered as novel candidates for PDAC therapy.

## RESULTS

### Aberrant OX1R expression in PDAC

### Human PDAC in TMA

**Figure 1:**
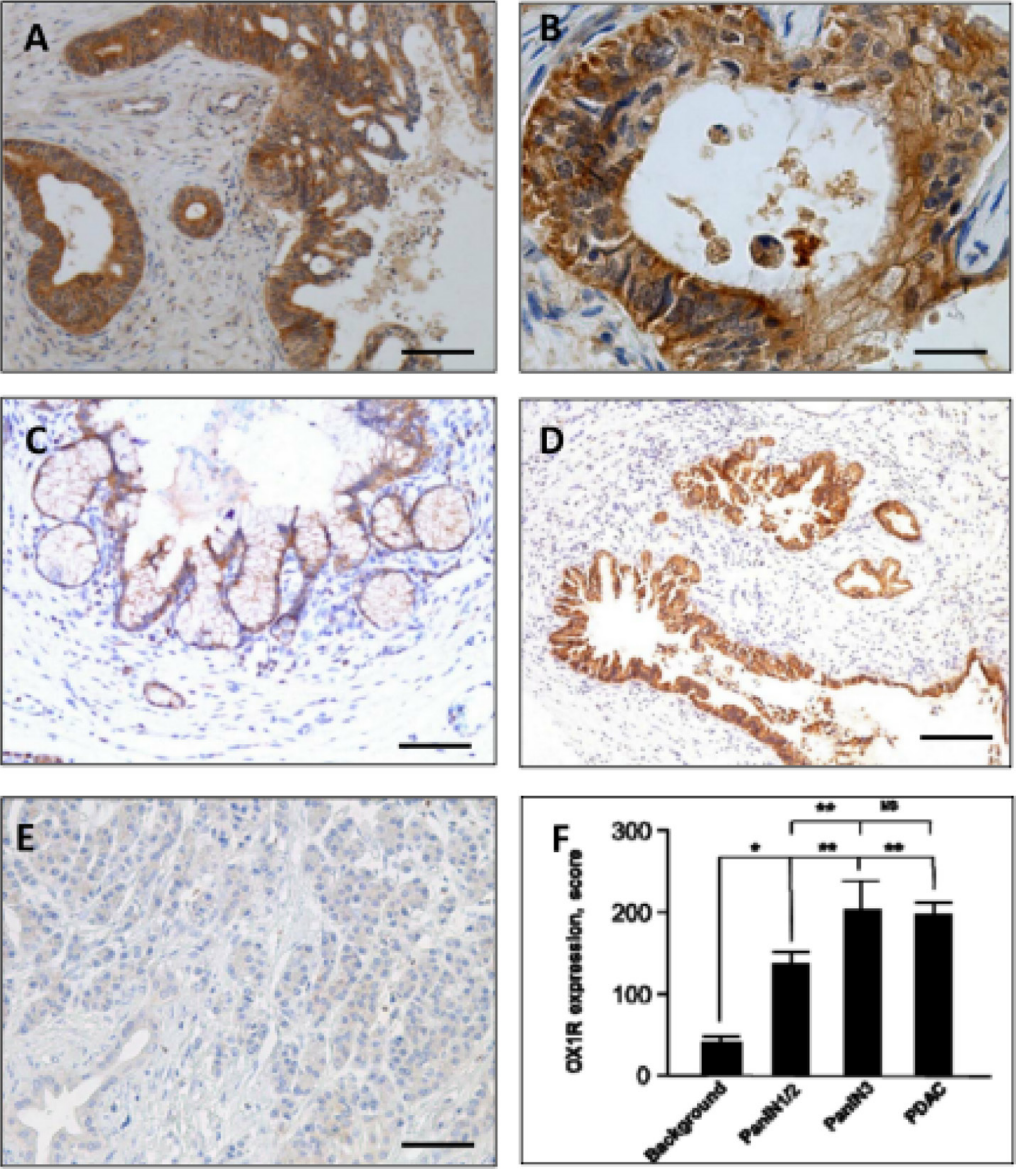
Immunohistochemical expression of the Orexin Receptor (OX1R) by PDAC (**A** and **B**), PanIN lesions (**C** and **D**) and normal pancreas (**E**) - OX1R is strongly expressed by tumor cells in PDAC, but is not detected in the surrounding stroma (A); at higher magnification, the staining is located to the membrane (arrows, B) and cytoplasm, and scored at 300 (intensity 3 on 100% of tumor cells, see “Materials and Methods”). OX1R was not detected in the normal pancreas (E) either in normal duct and acinar cells. OX1R was faintly expressed in a PanIN-1/2 lesion (C) and strongly expressed in a PanIN-3 lesion (D). Bar = 200 μm for (A), 50 μm for (B), 120 μm for (C), 400 μm for (D) and 120 μm for (E). OX1R immunostaining is scored in panel (**F)**. ^*^p < 0.05; ^**^p < 0.005 and ns, non-significant.

Seventy primary PDAC (70/73; 96%) expressed OX1R by immunohistochemistry, as shown in Figure 1A and 1B. OX1R expression was mainly cytoplasmic and membranous. Scores ranged from 90 to 300 (median: 188). Only three tumors did not show any immunoreactivity for OX1R (3/73; 4%). OX1R expression in PDAC was not correlated with patient age, gender, disease recurrence, disease-free survival, overall survival, tumor size, TNM stage, lymph node metastasis, or tumor differentiation (Table 1).

**Table 1:** Correlation of OX1R expression and main histopathological and clinical factors

	OX1R > median	OX1R < median	*p* value
T1 or T2	4	7	NS
T3	32	27	
NO	8	10	NS
N1	28	24	
Tumor size <30 mm	16	15	NS
Tumor size ≥30 mm	20	19	
Well-differentiated	19	17	NS
M or P differentiated	18	17	
Recurrence	29	24	NS
No recurrence	7	10	
Dead of disease	28	27	NS
Alive	8	7	

NO: no lymph node metastasis; N1: presence of lymph node metastasis; M or P: moderately or poorly.

### Human PanIN and normal pancreas

OX1R was expressed in 20/20 (100%) PanIN lesions. Its expression was higher in PanIN-3 as compared to PanIN-1/2 (*p* < 0.005; Figure 1C and 1D). In contrast, no OX1R immunodetection was observed in normal exocrine pancreas, including acinar and ductal cells; OX1R was restricted to islets in normal pancreas (Figure 1E and 1F).

### OX1R expression in AsPC-1 cell line

**Figure 2:**
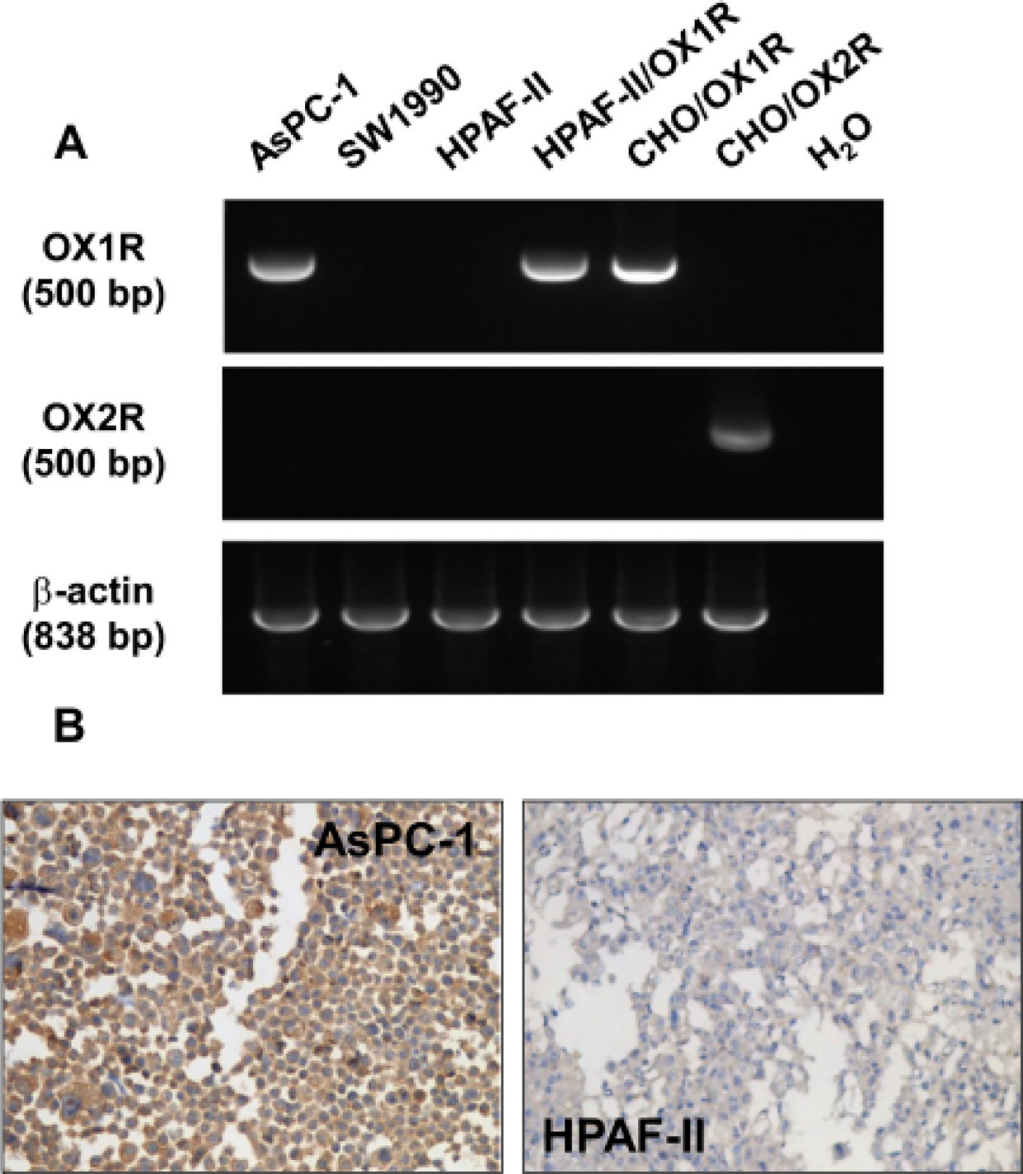
Expression of OX1R in PDCA cells – (**A**) shows RT-PCR analysis of OX1R (top panel) or OX2R (middle panel) mRNA from AsPC-1 cells, SW1990 cells, parental HPAF-II cell, HPAF-II cells expressing recombinant OX1R, CHO/OX1R cells, and CHO/OX2R cells. Controls are shown in the last lane (H_2_O) in absence of DNA template. RT-PCR analysis of β-actin mRNA was used as control (bottom panel). (**B**) shows the immunostaining of OX1R in paraformaldehyde-fixed and paraffin-embedded section from pellets of AsPC-1 cells (left panel) and HPAF-II cells (right panel) cultured in standard medium in the presence of FCS.

As shown in Figure 2A, an amplified single specific 500 bp PCR product corresponding to OX1R transcript was detected in the AsPC-1 cell line. CHO cells expressing recombinant OX1R receptor were used as control. No OX1R transcript was detected in the SW 1990 and HPAF-II cancer cell lines. As shown in Figure 2A, no mRNA could be detected for the other orexin receptor subtype, OX2R, in any cell lines tested as compared to control recombinant CHO/OX2R cells.

These data are in full agreement with the immunostaining data for OX1R in AsPC-1 and HPAF-II cell lines included in cell-blocks: specific OX1R immunodetection was observed in AsPC-1 cell membranes whereas no OX1R expression could be seen in the HPAF-II cell line (Figure 2B)

### Orexin-A effect *in vitro* cell line models

**Figure 3:**
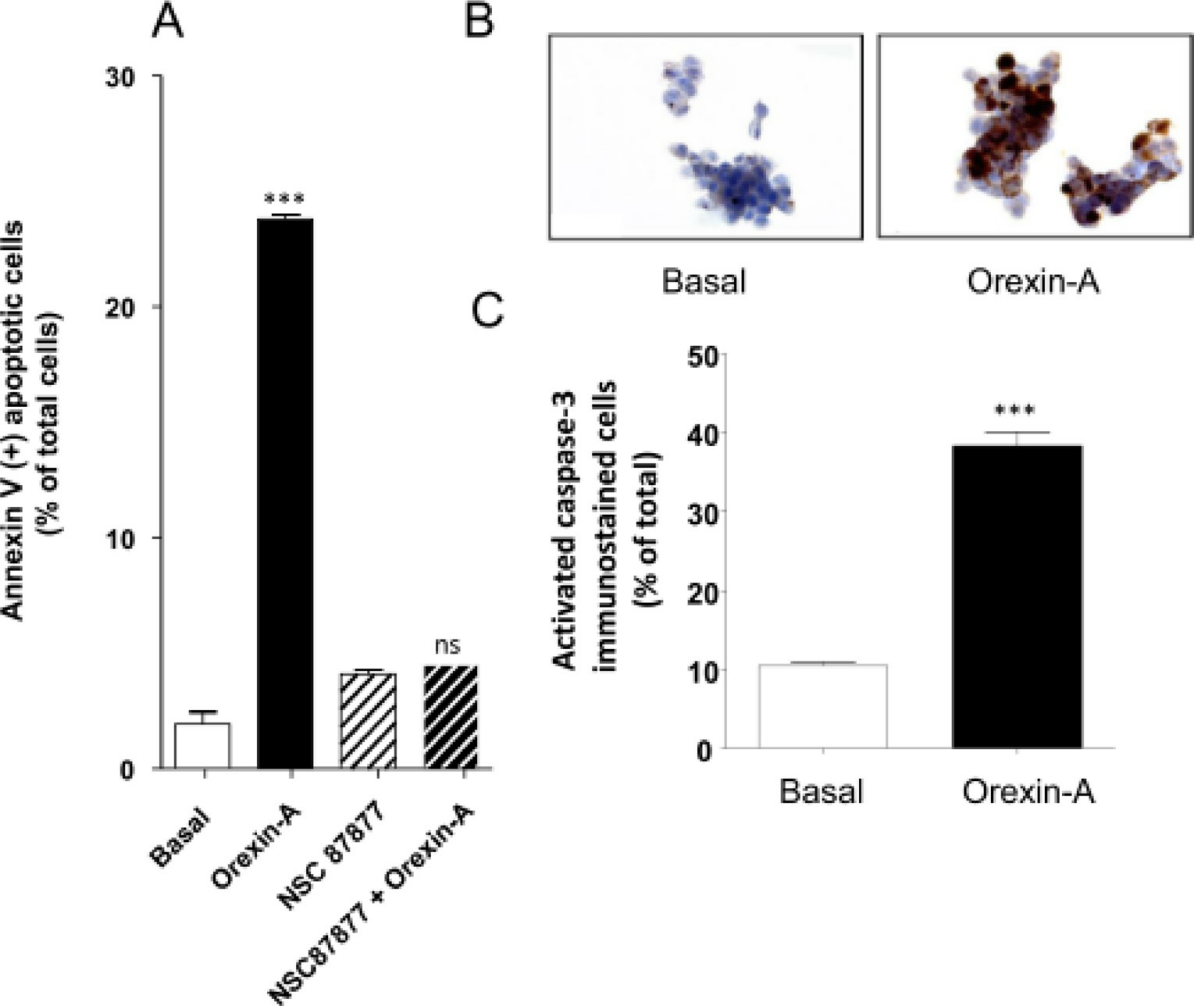
Effect of orexin-A on apoptosis in AsPC-1 cells - (**A**), SHP-2 protein tyrosine phosphatase inhibitor, NSC-87877, blocks orexin-induced apoptosis. AsPC-1 cells were challenged with (black bars) or without (white bars) 1 μM orexin-A for 48 hr in the absence or presence of NSC-87877 (50 μM). Apoptosis was measured by determination of annexin V-PE binding, and results are expressed as the percentage of apoptotic cells; (**B**) and (**C**). Indirect immunostaining of activated caspase-3 in AsPC-1 cells in the presence or absence of orexin-A. Paraformaldehyde-fixed AsPC-1 cells were challenged with (orexin-A) or without (basal) 1 μM orexin-A for 48 hr. Activated caspase-3 immunostaining is shown in B and scored in C. Results are means ± SE of three separate experiments. ^***^p < 0.001; ns, non-significant.

Orexin-A induced a drastic inhibition of cellular growth of AsPC-1 cells, associated with the induction of mitochondrial apoptosis, characterized by recruitment of the tyrosine phosphatase SHP-2 and followed by the activation of caspase-3. One μM orexin-A induced a strong increase of annexin-V positive AsPC-1 cells (24.3% ± 1.4) as compared to untreated cells (3.8% ± 1.9) (Figure 3A). In the presence of the specific SHP-2 inhibitor, NSC 87877, the orexin-A-induced apoptosis was totally abolished (Figure 3A), in agreement with the involvement of a SHP-2-dependent apoptosis signaling pathway. No significant difference was observed in the NSC 87877-treated AsPC-1 cells in the presence (4.8% ± 0.5) or absence of 1 μM orexin-A (4.6% ± 0.9). Moreover, 1 μM orexin-A induced the cleavage and activation of caspase-3, as seen by immunohistochemistry, whereas no activated caspase-3 was detected in untreated cells (Figure 3B). The quantification of the staining demonstrated that orexin-A induced a 4-fold increase in activated caspase-3 as compared to basal conditions (Figure 3C).

**Figure 4:**
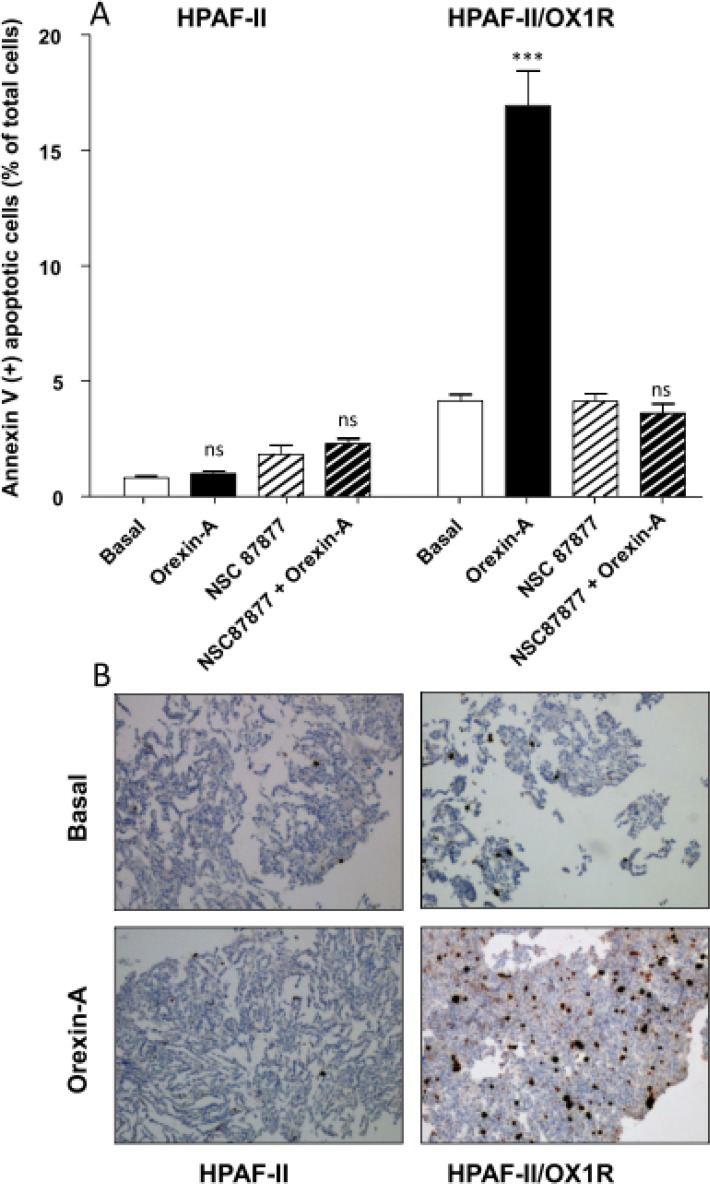
Effect of orexin-A on apoptosis in OX1R expressing recombinant OX1R/HPAF-II cells - (**A**), Parental HPAF-II and recombinant OX1R/HPAF-II cells were challenged with (black bars) or without (white bars) 1 μM orexin-A for 48 hr in the absence or presence of the SHP-2 protein tyrosine phosphatase inhibitor, NSC-87877 (50 μM). Apoptosis was measured by determination of annexin V-PE binding. Results are expressed as the percentage of apoptotic cells, and are the means ± SE of three separate experiments. ^***^p < 0.001; ns, non-significant; (**B**), Paraformaldehyde-fixed HPAF-II cells and recombinant OX1R/HPAF-II cells were challenged with or without (Basal) 1 μM orexin-A for 48 hr. Indirect immunostaining of activated caspase-3 in parental HPAF-II cells (left panels) and recombinant OX1R/HPAF-II cells (right panels) in the presence (bottom panels) or tabsence (top panels) of orexin-A is illustrated in B.

**Figure 5:**
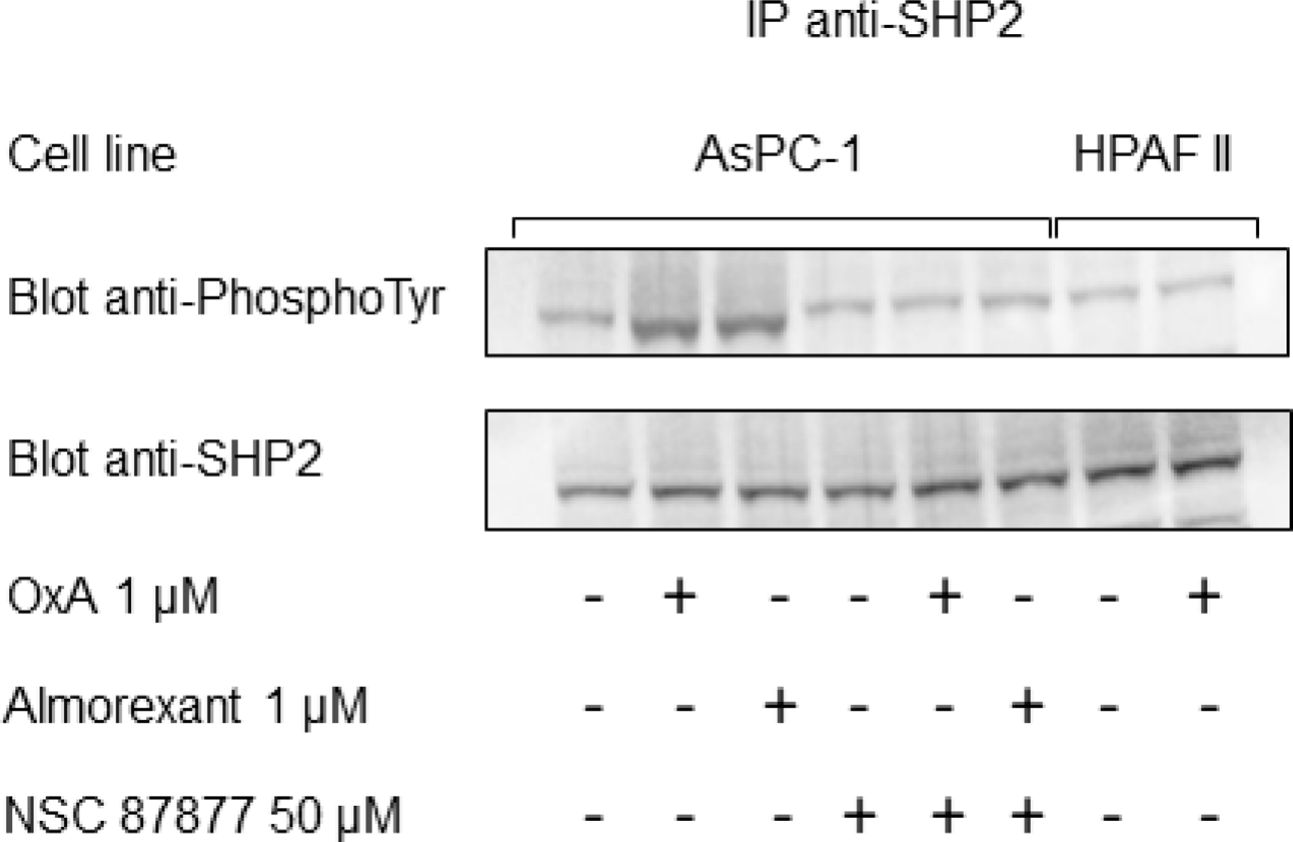
Orexin-A and almorexant promote tyrosine phosphorylation of SHP2/OX1R complex AsPC-1 cells preincubated or not with the SHP2 inhibitor NSC-87877 (50 μM) were challenged for 5 min without or with 1 μM orexin-A or 1 μM almorexant. In parallel, HPAF-II cells were also challenged for 5 min without or with 1 μM orexin-A. After cell lysis, anti-SHP2 antibodies were used to obtain anti-SHP2 immunoprecipitates (IP) from 500 μg of lysate protein. Western blot analysis was then performed using the antibodies anti-PhosphoTyrosine and anti-SHP2.

In order to demonstrate the specific proapoptotic role of OX1R in PDAC, we expressed recombinant OX1R in HPAF-II cells, which do not express this receptor. In parental HPAF-II cells, treatment with 1 μM orexin-A did not induce apoptosis (Figure 4A). Inversely, treatment of recombinant HPAF-II expressing OX1R cells with 1 μM orexin-A resulted in the strong induction of cellular apoptosis as indicated by 17.8% ± 2.4 annexin-V positive apoptotic cells (Figure 4A) compared to 2.1% ± 0.4 in untransfected cells. In addition, NSC87877 totally abolished orexin-A-induced apoptosis in OX1R-expressing HPAF-II cells (Figure 4A), whereas this inhibitor had no effect on the parental cells. Similarly, orexin-A induced a strong caspase-3 activation in recombinant OX1R/HPAF-II cells while no activation was observed in the parental cells (Figure 4B). As previously demonstrated in colon cancer, orexin-A induced both OX1R phosphorylation and SHP2 recruitment by OX1R leading to apoptosis process [16]. After immunoprecipitation of SHP2/OX1R complex expressed in AsPC-1 cells, we observe that orexin-A promotes tyrosine phosphorylation of OX1R. However, in the presence of NSC87877 inhibitor, orexin-A was unable to stimulate tyrosine phosphorylation of receptor. Inversely, in HPAF II cells which did not express OX1R, no tyrosine phosphorylation was observed in the presence of 1 μM orexin-A (Figure 5).

These data strongly suggest that the mechanism involved in orexin-A-induced apoptosis in pancreatic cancer cells was similar in colon cancer cell lines as previously described [8, 16].

### Orexin-A effect in preclinical models

### AsPC-1 xenografts

**Figure 6:**
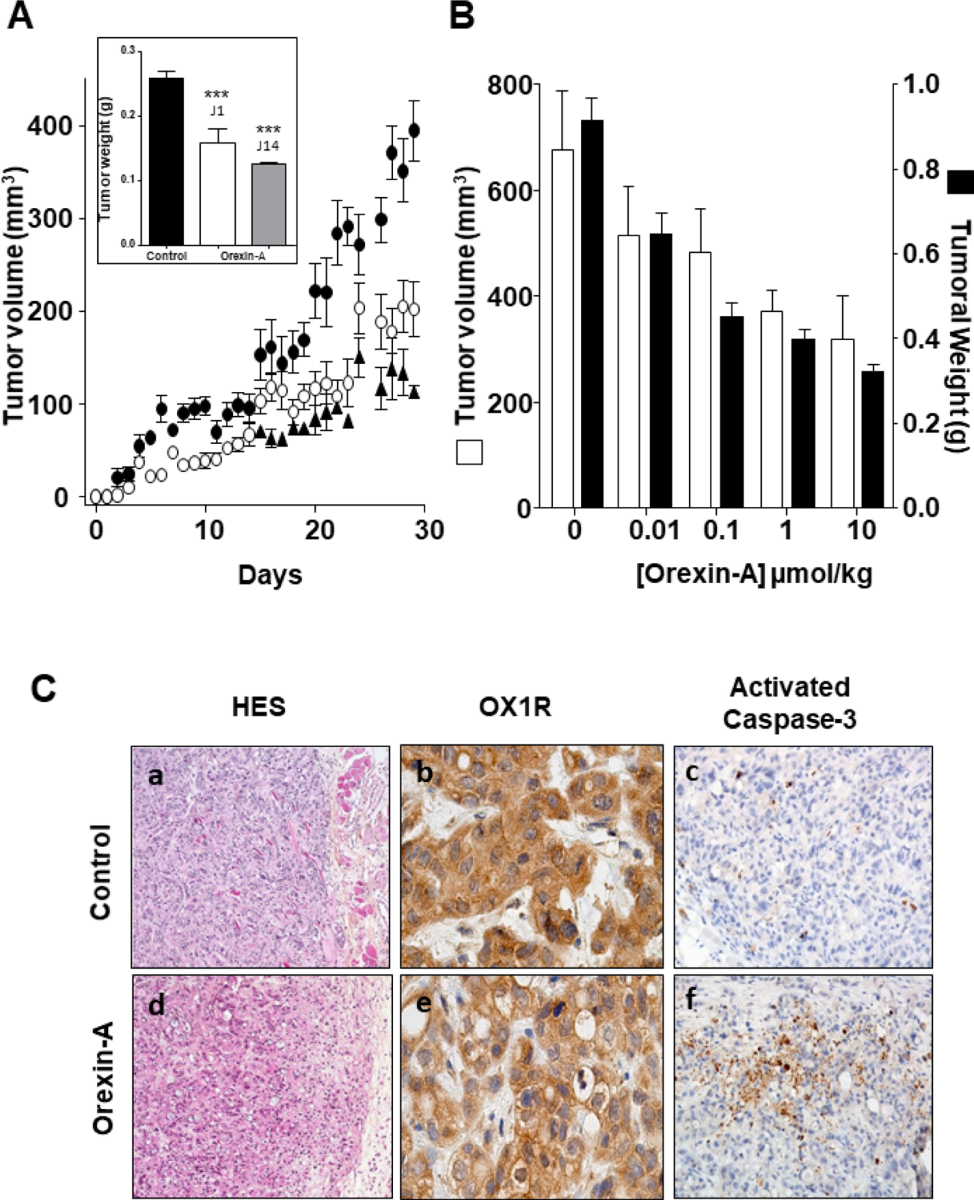
Effect of daily inoculation of orexin-A on the growth of tumors developed by xenografting human PDCA cells in nude mice - AsPC-1 cells were inoculated in the flank of nude mice at day 0 Mice were injected daily intraperitoneally with 100 μl of orexin-A solutions starting at day 0 (○) or day 14 (▲) or with 100 μl of PBS (●) for controls. (**A**) The daily treatment corresponded to 1 μmoles of orexin-A/Kg. Inset represents the tumor weight measured at the end of the experiment after the mice were sacrificed; (**B**) Mice received 0.01, 0.1, 1 or 10 μmoles of orexin-A/Kg. After 30 days of treatment, mice were sacrificed and tumor volume and weight were then recorded. The developement of tumors was followed by caliper measurement. Data are the means ± SE of 6 tumors in each group. ^***^p < 0.001 versus control. Indirect immunostaining of activated caspase-3 in xenografted AsPC-1 tumors resected from nude mice (**C**) Paraformaldehyde-fixed xenografted AsPC-1 tumors from nude mice treated daily (panels d, e and f) by intraperitoneal injections with 1 μmoles/Kg orexin-A or not (panels a, b and c). After necropsy, tumors were resected. Formalin-fixed paraffin-embedded tumours were cut in 3 μm sections, which were either stained with hematoxylin-eosin or used for immunohistochemistry. Orexin-A induced tumoral cell death (panel d), as detected by Hemalum Eosin Safran (HES) staining, which correlated with apoptosis induction assessed by strong immunostaining of activated caspase-3 after 30 days of orexin-A treatment; (panel f). OX1R immunostaining localisation was similar under control and orexin-A treatment conditions.

Daily intraperitoneal injection of orexin-A (1 μmol/kg) at day 0 in mice xenografted with AsPC-1 cells and up to the mice sacrifices resulted in a significant decrease in tumor volume (48.8%), as compared to untreated mice (Figure 6A). In addition, treatment with orexin-A started after AsPC-1 tumors were developed, 14 days after cell inoculation. Orexin-A (1 μmol/kg), rapidly and strongly reduced the volume of established tumors (Figure 6A). After animal sacrifice, tumors were resected and weighted. No differences were observed in the weight of tumors from orexin-A treated mice at day 0 and orexin-A treated mice at day 14 after cell inoculation (Figure 6A, insert). The effect of orexin-A on tumor volume was dose-dependent as a 30-day treatment with 0.01, 0.1, 1 and 10 μmoles orexin-A/kg decreased the tumor volumes by 34.4, 30.6, 46.7, and 52.8%, respectively (Figure 6B). These data correlated with tumor weight (Figure 6B). Hematoxylin and eosin staining of resected tumors revealed the same glandular differentiation in both treated and non-treated tumors (Figure 6C, panels a and d). OX1R immunostaining level was not affected by orexin-A treatment, suggesting that OX1R expression is not altered by chronic orexin-A treatment (Figure 6C, panels b and e). Furthermore, weak and intense staining of activated caspase-3 was observed in control (Figure 6C, panel c) and orexin-A treated (Figure 6C, panel f) mice, respectively.

### Patient-derived xenografts

**Figure 7:**
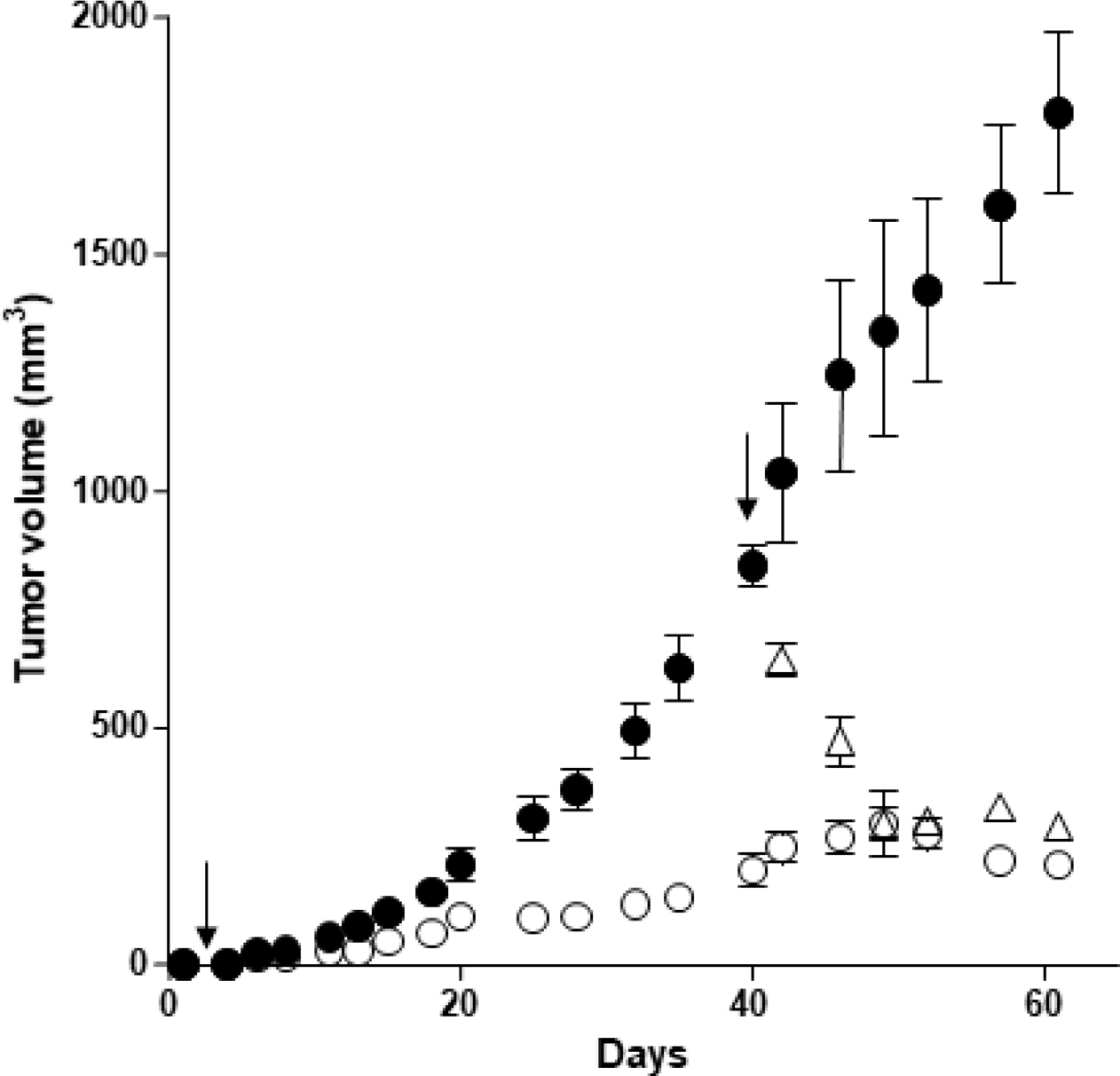
Effect of orexin-A inoculation on the growth of tumors developed by xenografting OX1R expressing pancreas cancer PDX cells in nude mice - PDX cells were inoculated (10^6^ cells per xenograft) in the flank of nude mice at day 0 Mice were injected (2 times/week) intraperitoneally with 100 μl of 1 μmoles of orexin-A/Kg solutions starting at day 1 (○) or day 40 (Δ) or with 100 μl of PBS (●) for controls. The development of tumors was followed by caliper measurement. Data are the means ± SE of 6 tumors in each group; ^***^p < 0.01 versus control.

In an effort to develop more reliable preclinical models, we have established a subcutaneous patient-derived xenograft (PDX) model. We have isolated cancerous cells from a human PDAC freshly resected. PDX cells were verified by the expression of OX1R (data not shown) and xenografted in mice. Daily intraperitoneal injection of orexin-A (1 μmol/kg) at day 0 in mice xenografted with PDX cells and up to the mice sacrifices resulted in a significant decrease in tumor volume (80%), as compared to untreated mice (Figure 7). In addition, treatment with orexin-A started after PDX tumors were developed, 40 days after cell inoculation. Orexin-A (1 μmol/kg), rapidly and strongly reduced the volume of established tumors (Figure 7).

### HPAF-II xenografts

As mentioned above, we demonstrated the specific inhibitory effect of OX1R on tumor growth in HPAF-II/OX1R xenografted in nude mice (Supplementary Figure 1). Daily treatment with 1 μmol orexin-A/kg of mice xenografted with parental HPAF-II cells was unable to promote tumor growth inhibition. When mice were xenografted with recombinant HPAF-II/OX1R this treatment induced 65% inhibition of tumor development. Xenografted nude mice treated with orexin-A after 14 days of tumor growth showed significant reduction in tumor volumes. Orexin-A treatment promoted a 3.5-fold caspase-3 expression by immunohistochemistry in tumors from recombinant HPAF-II/OX1R xenografted nude mice as compared to tumors from parental HPAF-II cells.

### Orexin-A effect in tumor explant models

**Figure 8:**
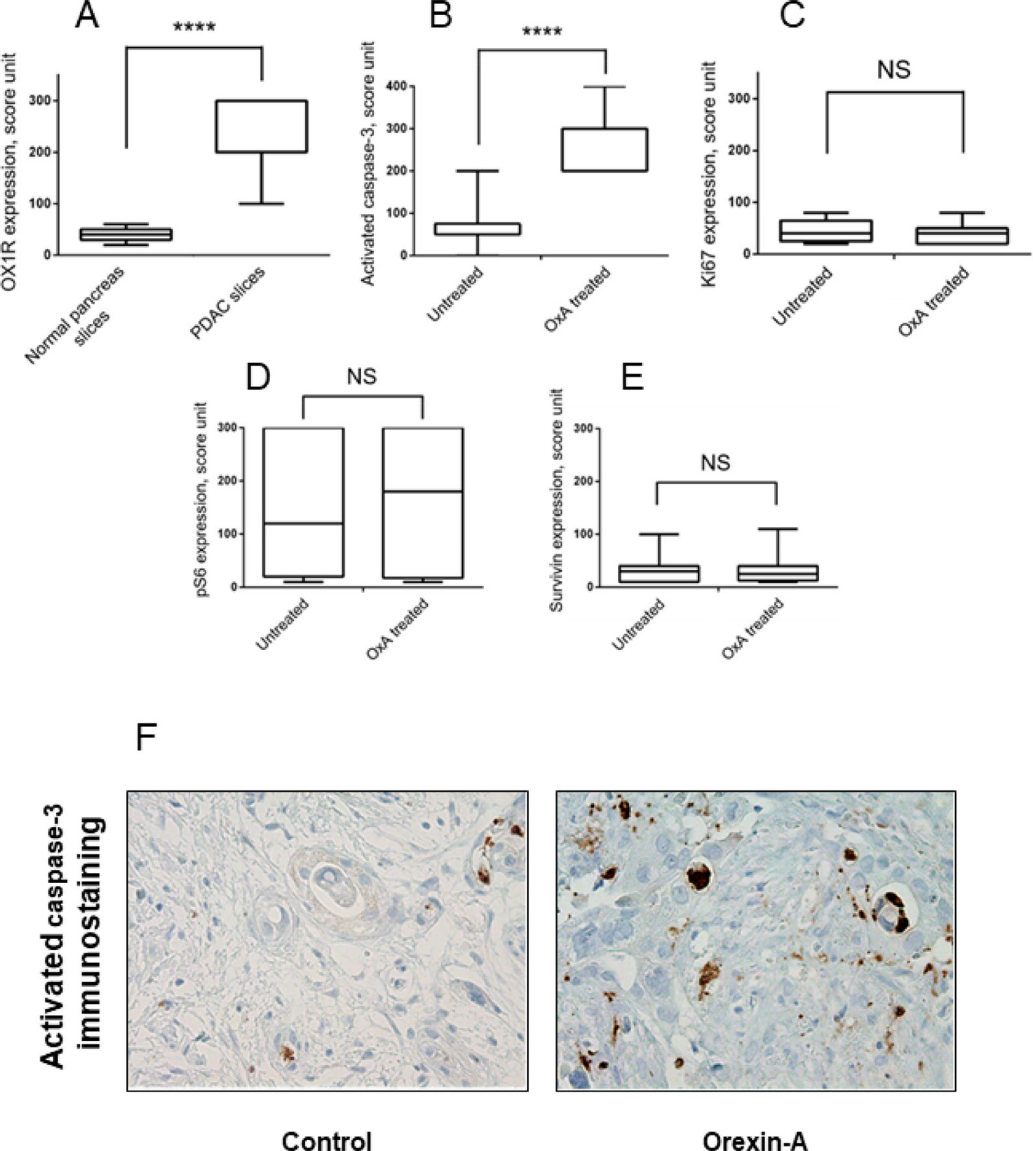
Effect of orexin-A on whole tumor tissue samples from patients with PDAC Panel **A** shows the score of OX1R expression in PDAC slices as compared to normal pancreas slices analysed by IHC. ^***^p < 0.0001 versus normal pancreas. The slice were treated for 24 hours with or without 1 μM orexin-A in triplicates and fixed overnight in formalin and then embedded in paraffin. Serial 3 μm thick sections were cut from the slices, stained by H&E or used for immunohistochemical staining to assess activated caspase-3 (**B**), Ki67 (**C**), pS6 (**D**) or surviving (**E**). Tissue quality was assessed by a pathologist (AC). If tissue integrity was not maintained over time (>20% necrosis induction), tissues were discarded. Graphs display protein expression scoring of the IHC analysis of samples from 11 PDAC patients treated *ex vivo* with orexin-A were represented in panels B, C, D and E. ^***^p < 0.001 versus untreated, NS, no significant. Panel **F** represents a pancreatic ductal carcinoma after 24 h of slice culture with (orexin-A, panel right) or without 1 μM orexin-A (control, left).

Significant OX1R immunostaining was observed in PDAC slices as compared to normal exocrine pancreas (score 227 *vs* 40; *p* < 0.0001) (Figure 8A). We observed, in slices treated by orexin-A, an increase of activated caspase-3 immunostaining in tumor cells as compared to the control slices without orexin-A treatment (Figure 8B and 8F and Table 2). Moreover, orexin-A treatment did not impact the cell proliferation (Figure 8C), neither ERK/MAPK and mTor signaling pathway (Figure 8D) nor surviving expression (Figure 8E). Taken all together, these results demonstrate that the OX1R/orexin-A pathway plays a crucial role in tumor growth inhibition.

**Table 2:** OX1R expression and effect of orexin-A on whole tumor tissue samples from patients with PDAC

	Normal pancreas slices	PDAC slices	*p* value
OX1R expression	40 ± 5	227 ± 23	0.0001
	**Untreated**	**OxA treated**	***p* value**
Activated caspase 3	67 ± 19	267 ± 24	0.0001
Ki67 expression	44 ± 8	40 ± 8	0.6912
pS6 expression	154 ± 53	165 ± 61	0.9115
Survivin expression	33 ± 9	35 ± 12	0.8964

OX1R expression, activated caspase-3, Ki67, pS6 and survivin immunostaining are scored. Results are the means ± SE of 11 independant patients.

### The Ca^2+^ pathway orexin receptors antagonist, almorexant acts also as an anti-tumoral agent

**Figure 9:**
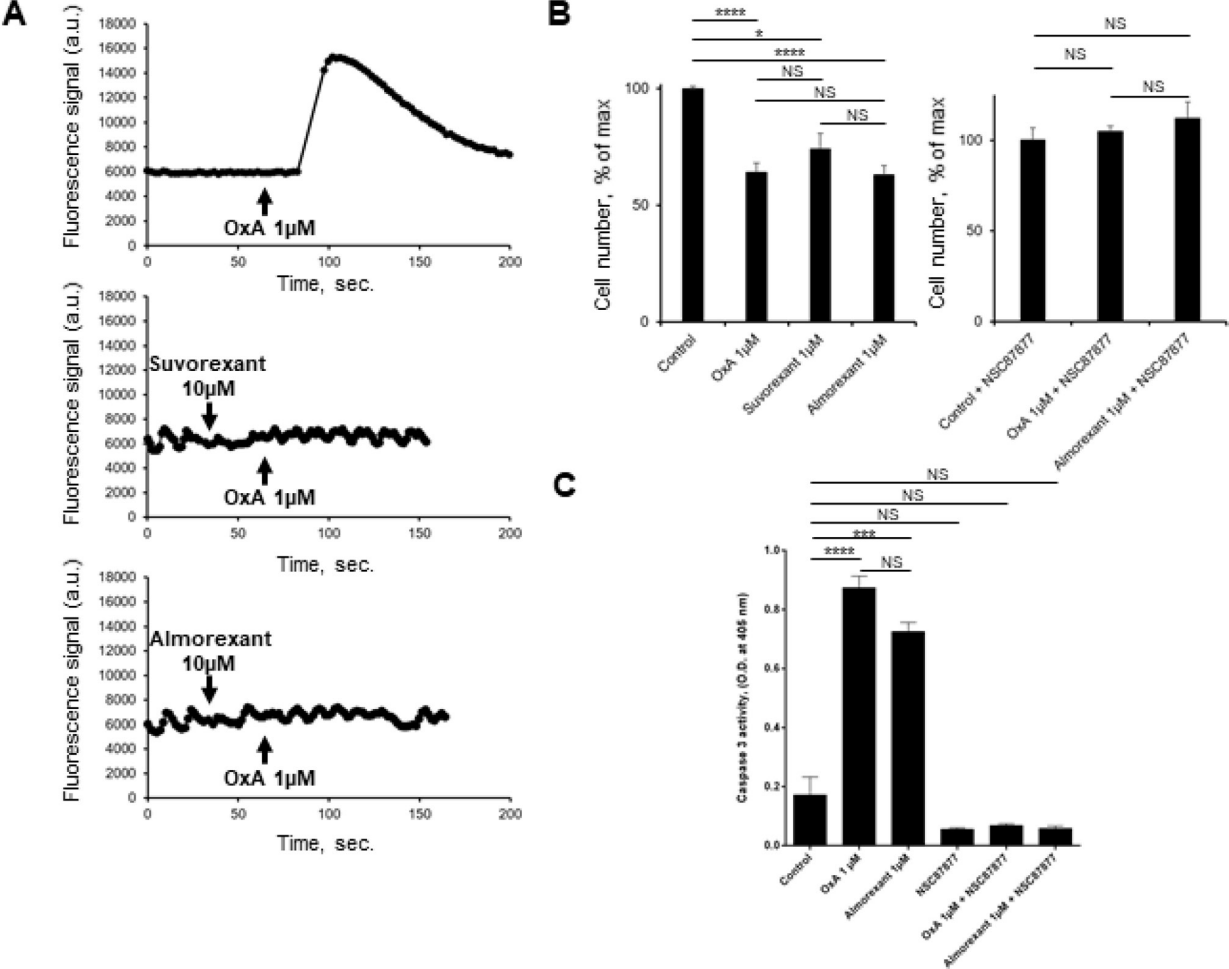
Effect of almorexant and suvorexant on intracellular Ca^2+^ release, cell growth and caspase-3 activity (**A**) Intracellular Ca^2+^ production was detected in HEK-293 cells expressing recombinant native OX1R using Fluoforte Calcium Assay Kit (Enzo Life Sciences, NY, USA). Cells were challenged with 1 μM of OxA (top panel) or 1 μM OxA after preincubation with 10 μM suvorexant (middle panel) or 10 μM almorexant (bottom panel). (**B**) AsPC-1 cells were incubated for 48 h with OxA or almorexant or suvorexant in the presence (right panel) or in the absence (left panel) of 50 μM NSC87877. (**C**) colorometric Caspase-3 activity detection at 405 nm in AsPC-1 cells incubated with 1 μM OxA or 1 μM almorexant in the presence or in the absence of 50 μM NSC87877. ^*^p < 0.05, ^***^p < 0.001, ^****^p < 0.0001 and NS, no significant.

The pharmacology of orexin/orexin receptors had widely developed in orexin-regulation of sleep background [22]. Recently, several orexin antagonists including almorexant and suvorexant had been described in order to control insomnia. In the present work, we have investigated the impact of almorexant and suvorexant, on their abilities to promote pro-apoptotic and anti-tumoral effects. As expected, suvorexant and almorexant totally blocked the intracellular Ca^2+^ signal pathway. Indeed, incubation of 1 μM OxA with HEK-OX1R cells which expressed recombinant OX1R led to a massive increase of cytosolic Ca^2+^ mobilization (Figure 9A). In contrast, when HEK-OX1R cells were incubated with 1 μM OxA and 10 μM suvorexant or almorexant, the Ca^2+^ mobilization was totally inhibited (Figure 9A). Surprisingly, 1 μM of suvorexant or almorexant inhibited the cellular growth of AsPC-1 cells (Figure 9B). However, almorexant is more potent than suvorexant in the AsPC-1 cell growth inhibition. Moreover, 1 μM almorexant presents the similar effect than 1 μM orexin-A inducing 37 ± 4% and 36 ± 4% cell death, respectively (Figure 9B). based on these observations, only almorexant will be used in next experiments. Almorexant-induced cell growth inhibition was totally abolished by NSC-87877 cell pre-treatment (Figure 9B). This effect was associated to almorexant-induced apoptosis, as shown in Figure 8C almorexant stimulates caspase-3 activity in AsPC-1 cells similarly to orexin-A-induced caspase-3 activation (Figure 9C). As expected, cells pretreatment with NSC87877 inhibitor totally reversed this effect induced by almorexant and orexin-A (Figure 9C). Moreover, almorexant induced tyrosine phosphorylation of OX1R after immunoprecipitation of SHP2/OX1R complex in AsPC-1 cells. Almorexant-induced tyrosine phosphorylation of OX1R was totally inhibited in the presence of NSC87877 inhibitor (Figure 5). These data demonstrated that almorexant was able to induced apoptosis in pancreas cancer cells.

**Figure 10:**
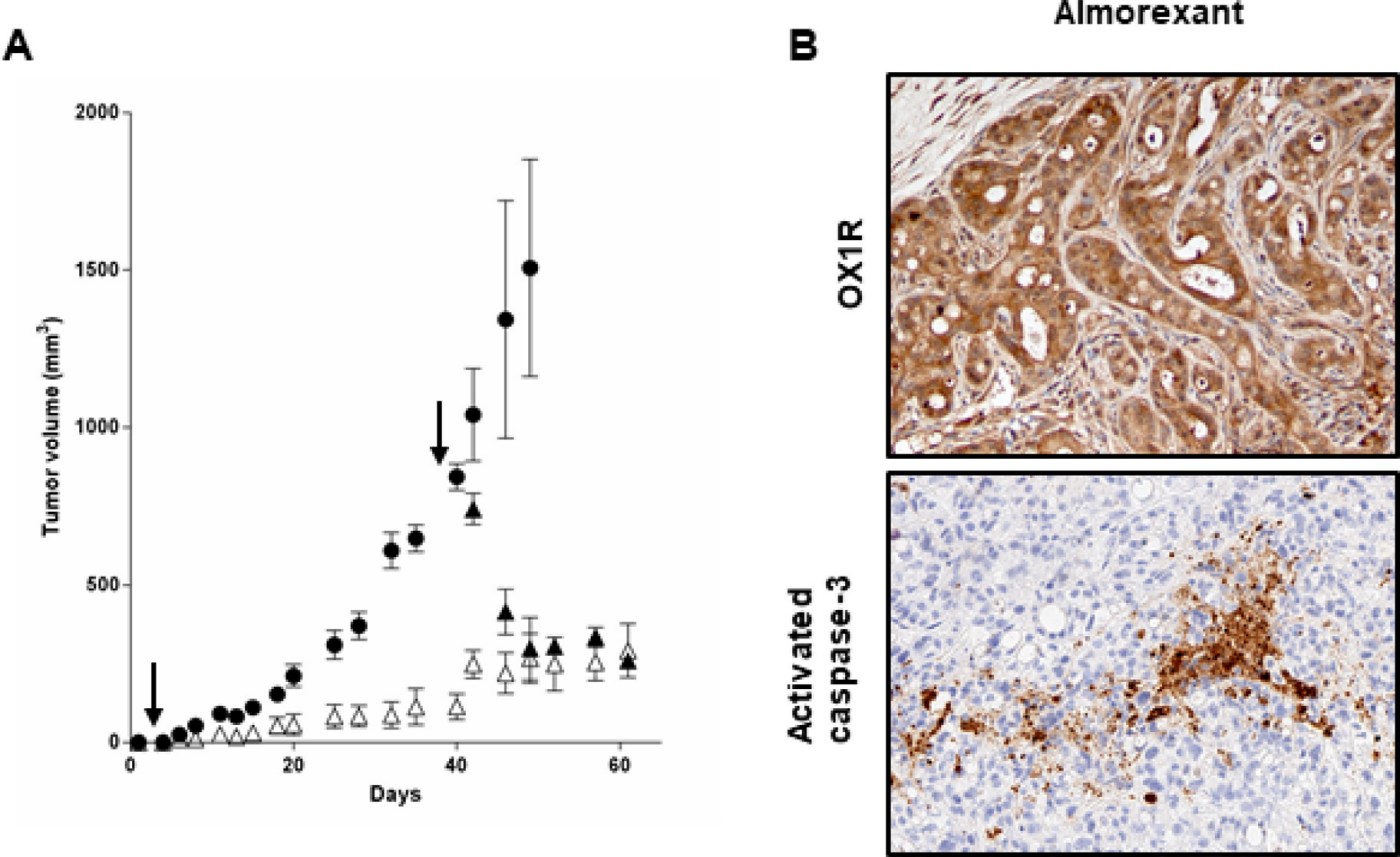
Effect of daily inoculation of almorexant on the growth of tumors developed by xenografting human PDCA cells in nude mice - (**A**) AsPC-1 cells were inoculated in the flank of nude mice at day 0. Mice were injected daily intraperitoneally with 100 μl of almorexant (1.8 μmol/kg) solutions starting at day 0 (Δ) or day 38 (▲) or with 100 μl of PBS (●) for controls. After 60 days of treatment, mice were sacrificed and tumor were then fixed. The development of tumors was followed by caliper measurement. Data are the means ± SE of 6 tumors in each group. ^***^p < 0.001 versus control. (**B**) Indirect immunostaining of OX1R (top) and activated caspase-3 (bottom) in xenografted AsPC-1 tumors resected from nude mice. After necropsy, tumors from nude mice treated by almorexant were resected and paraformaldehyde-fixed and then were cut and used for immunohistochemistry.

In this context, *in vivo* studies in preclinical model were investigated. Daily intraperitoneal injection of almorexant (1.8 μmol/kg) at day 0 in mice xenografted with AsPC-1 cells and up to the mice sacrifices resulted in a significant decrease in tumor volume (> 50%), as compared to untreated mice (Figure 10A). It should be noted that almorexant and orexin-A anti-tumoral effect were similar (Figure 6A and 10A). In addition, treatment with almorexant started after AsPC-1 tumors were developed, rapidly and strongly reduced the volume of established tumors (Figure 10A). OX1R expression revealed by immunodetection was not affected by almorexant treatment (Figure 10B).

## DISCUSSION

We reported that OX1R is aberrantly expressed in 96% of PDAC and 100% of PanIN, but not in normal acini and ducts adjacent to PDAC tissue although OX1R was expressed in islets. Orexin-A was able to promote apoptosis: 1) *in vitro*, in the PDAC cell line, AsPC-1; 2) *ex vivo*, in tissue culture derived from patients. Furthermore, orexins strongly reduce the development of tumors in nude mice xenografted with human PDAC cells derived from cell line or PDX.

It is well known that PDAC are highly malignant neoplasms characterized by their poor prognosis and weak response to treatments [23]. In this regard, the expression of OX1R in primary PDAC tumors represents an essential element for new therapies as demonstrated previously in colon cancer by our group. [24, 25]. Because apoptosis is a highly controlled process regulated by different cellular signaling events, proapoptotic drugs are currently considered as attractive candidates in anticancer targeted therapies [26, 27]. In the present work, we have demonstrated that the proapoptotic activity of OX1R correlates well with inhibition of cell growth in AsPC-1 pancreatic cell lines treated with orexin-A. The expression of recombinant OX1R in a given cell line (HPAF-II) is sufficient to promote orexin-mediated apoptosis, highlighting the intrinsic property of the orexin receptor [15, 17]. The proapoptotic properties of OX1R are mediated through recruitment of the tyrosine phosphatase SHP-2 [14, 16, 17] In our work, in the presence of NSC-87877, a potent inhibitor of SHP-2, the proapoptotic action of orexin-A was significantly reversed, confirming that SHP-2 activity is an essential step in OX1R-mediated apoptosis in PDAC.

*Ex vivo* experiments demonstrated that orexin-A was able to activate caspase-3 in cultured tumor tissue removed from several patients showing the potential impact in the clinical setting. In contrast, orexin-A has no effect on the expression of proliferation marker, Ki67, confirming that orexin has no anti-proliferative properties [15].

*In vivo* experiments showed that orexin-A slows AsPC-1 tumor growth in a dose-dependent manner between 0.01 and 10 μMoles/kg. In addition, orexin-A was able to decrease tumor volume from tumors established 14 days prior (see Figure 6). Moreover, orexin-A slows PDX tumor growth and was able to decrease tumor volume from tumors established 40 days prior (see Figure 7). It is well known that the trafficking of various GPCRs differs depending on the time and concentration of exposure to ligands [27]. After a prolonged or repeated interaction with agonists, the total number of cell surface receptors can decrease by downregulation [28–30]. Nevertheless, OX1R expression in xenografted tumors was not altered by orexin treatment, suggesting that the receptor is not downregulated by the presence of its ligand.

Based on the success of the somatostatin receptor in targeting neuroendocrine tumors, novel tracers for peptide receptors such as analogs for prostate and breast cancers or for neo-angiogenesis labeling, are currently emerging [31]. Considering that OX1R is widely expressed in PDAC, has high affinity for orexins, and is not expressed in non-tumoral tissues, the development of isotopic orexin probes that could be used for the detection of precancerous PanIN lesions or micro-metastases by medical imaging should be tested in the future.

Actually, it is well known that a lot GPCR specific ligands were able to activate independently different signaling pathways [32]. These ligands also termed ligand-biased could stabilize different conformations of GPCR leading to promote various signaling pathways [32, 33]. An increasing number of studies described several non-peptidic chemical OX1R antagonists belonging to SORA (Single Orexin Receptors antagonist) and DORA species [22]. These molecules had been developed to inhibit the canonic intracellular calcium mobilization pathway related to the regulation by orexins of sleep control [22]. Suvorexant is the first agent to be approved in this new class of medication [22] and gained US Food and Drug Administration (FDA). In the present paper, we demonstrated that DORA such as suvorexant and more specifically, almorexant which are, as expected, full calcium pathway antagonists but are surprisingly, full proapoptotic pathway agonists suggesting that these molecules belong to ligand-biased family. Structure-function relationship analysis by ala-scanning of orexin-B have demonstrated that some residues of the peptide were able to discriminate between calcium and proapoptotic pathways [34]. Similarly, almorexant which bind to OX1R with the same affinity than orexin-A could distinguish these two signaling pathways suggesting the existence of two independent molecular activation processes in OX1R receptor. These observations indicate that OX1R antagonists which are used in insomnia treatment could be used in PDAC therapy.

In conclusion, we have compellingly shown that OX1R is aberrantly expressed in PDAC, and that its activation by orexins and almorexant result in strong apoptosis and consequent cell growth inhibition *in vivo*, *ex vivo* and *in vitro* models. Based on our results, OX1R represents a new attractive and specific mediator of apoptosis against PDAC. The validation of orexin receptor as a target will lead to the development of new pharmacological molecules having a strong impact in the diagnosis and treatment of patients with PDAC. One of the possible paths of new therapeutical approaches could be the use of ligand-biased of OX1R such as almorexant.

## MATERIALS AND METHODS

### Human surgical samples with PDAC, PanIN lesions and normal pancreas

Seventy-three patients with PDAC treated with surgery (pancreato-duodenectomy *n* = 61; left pancreatectomy *n* = 9; total pancreatectomy *n* = 3) from April 1997 to December 2004 were selected from the files of the Department of Pathology, Beaujon Hospital, Clichy, France. Charts from patients were retrospectively reviewed for clinical and pathological data. No patients received chemotherapy or radiation therapy preoperatively. The following data were recorded: age, gender, recurrence, disease-free survival (DFS) and overall survival (OS), tumor size, TNM stage, lymph node metastasis, differentiation. The studied population included 38 men and 35 women. The median age at surgery was 60 years (range 34–76). The tumor stage was T1 in 3 patients, T2 in 8 patients and T3 in 59 patients. The median tumor size was 30 mm (range 10–100 mm). Lymph node metastases were present in 52 patients. Tumors were well- (*n* = 36), moderately- (*n* = 22) or poorly- (*n* = 12) differentiated. The median follow-up was 677 days (range 142–4294). Fifty-five patients (78,6%) died of the disease during the time of the study. Tissue microarray (TMA) blocks were produced from representative paraffin blocks from the 73 PDAC using a tissue arrayer (Manual Tissue Arrayer-MTA1, Beecher Instruments, WI, USA). We also selected 10 normal pancreas, taken at a distance from tumors, without any pancreatitis or fibrosis lesions but with precancerous lesions (pancreatic intraepithelial lesions, PanIN) of low grade (PanIN-1 or PanIN-2; *n* = 16) and high grade (PanIN-3; *n* = 4) grade of dysplasia. *For Ex Vivo* human PDAC slices cultures, the effects of orexin-A were tested on human freshly surgically resected PDAC (*n* = 11). After pathological evaluation a tumor sample was sliced using a Leica Tissue Slicer (VT1200 S, Leica Biosystems Nussloch GmbH, Germany) into 300 μm-thick slices cultured in inserts already placed in 6-well plates with William's E medium, complemented with components including foetal calf serum, glucose, gentamicin and HEPES, under normoxic conditions. The use of human material was approved by the Institutional Review Board (CEERB GHU Paris Nord N°IRB12-059 and 12-033).

### Cell lines cultures

3 PDAC cell lines (AsPC-1, HPAF-II and SW 1990) from the American Type Culture Collection (ATCC, Manassas, VA) were grown according ATCC recommendations. The HPAF-II/OX1R, CHO/OX1R and CHO/OX2R cell lines, expressing recombinant human OX1R, were obtained as previously described [16]. The cell lines were incubated in the presence or absence of 1 μM orexin-A (GL Biochemicals, Shangaï, China) or suvorexant or almorexant (MedChemExpress, Sollentuna, Sweeden).

### Tumorigenicity assay in nude mice xenografts

AsPC-1, HPAF-II and HPAF-II/OX1R cells were inoculated subcutaneously into the flank of anesthetized mice as previously described [8]. In an effort to develop more reliable preclinical models, we have established a subcutaneous patient-derived xenograft (PDX) model. Tumoral cells isolated from a human pancreatic cancer were inoculated into the flank of mice. Tumor development was followed by caliper measurements in 2 dimensions (L and W), and the volume (V) of the tumor was calculated [35, 36]. Orexin-A or almorexant was administered by intraperitoneal injections, starting the day of cell lines subcutaneous inoculation or 14 days (AsPC-1 cells) or 40 days (PDX cells) after this date when tumours were established. Control mice received PBS. After necropsy, tumors were then resected, weighted and analyzed.

### Immunohistochemical procedures

Immunohistochemistry was performed on formalin-fixed paraffin-embedded normal pancreas, PanIN lesions, tumor tissue (from human tissue-microarrays, human tissue slices and mice xenografts) and on cell lines in pellets fixed in formalin and embedded in cell blocks (Shandon Cytoblock; Thermo Scientific; USA), with an automated immunohistochemical stainer according to the manufacturer's guidelines (automate BOND, Leica Microsystems). Slides were immunolabeled with antibodies against OX1R (Life Technology, PA5-33837, polyclonal rabbit, 1/100), activated caspase-3 (Abgent, E87-77, polyclonal rabbit, 1/100) or Ki-67 (DAKO, clone MIB-1, monoclonal mouse, 1/100). OX1R evaluation was performed in human normal pancreas, PanIN lesions, PDAC TMA, PDAC slices, in xenografted tumors and in cell lines by calculating a score (0–300) obtained by multiplying the intensity (negative, 0; weak, 1; moderate, 2; and strong, 3) by the percentage of stained cells. Internal positive controls consisted of normal pancreatic islets while the HEK/hOX1R cell line served as an external positive control. The evaluation of apoptosis was performed in PDAC slices, tumor cell lines and xenografted tumours by evaluation of the percentage of caspase-3 positive tumour cells. External positive controls consisted of normal lymph nodes. The evaluation of Ki-67 was performed in PDAC slices by evaluation of the percentage of Ki-67 positive tumor cells. External positive controls consisted of normal lymph nodes.

### Immunoprecipitation of SHP2

AsPC-1 or HPAF-II cells were pretreated 24 h without or with 50 μM SHP1/2 inhibitor NSC-87877. Semiconfluent cells were then treated with 1 μM orexin-A or 1 μM almorexant in fresh culture medium at 37° C for 5 min. Cells were collected and lysed in 50 mM Tris-HCl buffer pH 7.4 containing 0.25% Na deoxycholate, 150 mM NaCl, 1% Nonidet P-40 and 1 mM EGTA. Proteins (500 μg) were then incubated with 2 μg of anti-SHP2 antibodies overnight at 4° C. Protein immunoprecipitation was performed according to the manufacturer's instructions using the Seize protein G immunoprecipitation kit (Pierce, Rockford, IL, USA). Then, immunoprecipitated proteins suspended in Laemmli buffer were loaded onto a 10% SDS-polyacrylamide gel, transferred to a nitrocellulose membrane, and immunoblotted with anti-PhosphoTyrosine (1:1000 dilution) or anti-SHP2 (1:1000 dilution) antibodies. Immunocomplexes were revealed with secondary peroxidase conjugated antibodies, using a chemiluminescent kit.

### RT-PCR assays

RNA was extracted from cultured cells lines including AsPC-1, SW 1990, HPAF-II, HPAF-II/OX1R, CHO/OX1R and CHO/OX2R by using RNeasy^®^ Mini Kit (Qiagen). PCR amplifications using OX1R sense primer (5′-CCTGTGCCTCCAGACTATGA-3′) and OX1R antisense primer (5′-ACACTGCTGACATTCCATGA-3′), OX2R sense primer (5′-TAGTTCCTCAGCTGCCTATC-3′) and OX2R antisense primer (5′-CGTCCTCATGTGGTGGTTCT-3′) or β-actin sense primer (5′-ATCTGGCACCACACCTTCTACAATGAGCTGCG-3′) and β-actin antisense primer (5′-CGTCATACTCCTGCTTGCTGATCCACATCTGC-3′) were carried out as previously described [15].

### Quantification of apoptotic cells by annexin V labelling

AsPC-1, SW 1990, HPAF-II and HPAF-II/hOX1R cells (seeded at 5 × 10^4^ cells/well) were grown as described above. The culture medium was then replaced every 24 hr with fresh medium with or without 1 μM orexin-A or almorexant in the presence or in the absence of the SHP-2 inhibitor, NSC-87877 (50 μM) (Calbiochem, VWR International SAS, France). After 48 hr, apoptotic cells were determined using the Guava Nexin^TM^ kit (Guava Technologies, Hayward, CA, USA [15]. Results are expressed as the percentage of apoptotic phycoerythrin-labelled Annexin V (Annexin V-PE) positive cells and are the means of 3 independent analyses.

### Caspase-3 activity detection

AsPC-1 cells were pretreated 24 h without or with 50 μM SHP1/2 inhibitor NSC-87877. 5.10^6^ semiconfluent cells were then treated with 1 μM orexin-A or 1 μM almorexant in fresh culture medium at 37° C for 24 h. Caspase-3 activity detection was performed according to the manufacturer's instructions using the caspase-3 assay colorimetric kit (#ab39401, Abcam, Paris, France). The caspase-3 activity measurement is based on spectrophotometric detection at 405 nm of the chromophore p-nitroaniline after cleavage by the activated caspase-3 from the labeled substrate DEVD-p-nitroaniline. Results are expressed as the optic density (O.D.) at 405 nm for 200 μg of protein for each sample and are the means of 3 independent analyses.

### Statistical analysis

Mann-Whitney non-parametric tests were performed to compare categorical with continuous variables where the number of categories was two. When the number of categories was greater than two, ANOVA (analysis of variance) tests were used instead. Data were analyzed with the GraphPad Prism 5.04 statistical software for Windows. All statistical tests were 2-sided. The critical level of statistical significance was set at *p* < 0.05.

## SUPPLEMENTARY MATERIALS FIGURE


