# Supplementary material for: In vitro, in vivo and ex vivo demonstration of the antitumoral role of hypocretin-1/orexin-A and almorexant in pancreatic ductal adenocarcinoma
Source: Oncotarget. 2018 Jan 9;9(6):6952–67. doi: 10.18632/oncotarget.24084 (PMC5805528; doi:10.18632/oncotarget.24084)
Supplement: Supplementary file 1 [file oncotarget-09-6952-s001.pdf]

In vitro, in vivo and *ex vivo* demonstration of the antitumoral role of hypocretin-1/orexin-A and almorexant in pancreatic ductal adenocarcinoma

SUPPLEMENTARY MATERIALS

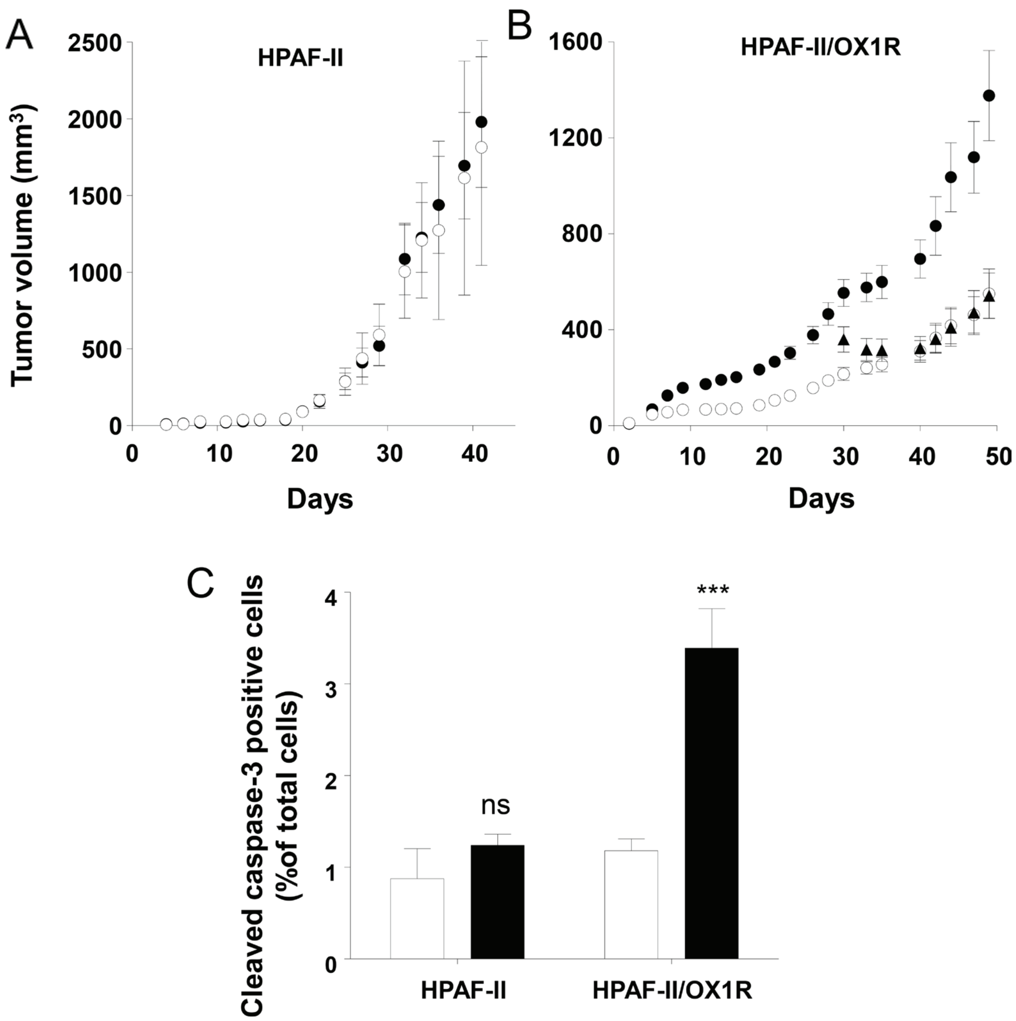

Supplementary Figure 1: Effect of daily inoculation of orexin-A on the growth of tumors developed by xenografting OX1R expressing recombinant HPAF-II cells in nude mice-Parental HPAF-II (**A**) or recombinant OX1R/HPAF-II/cells (**B**) were inoculated in the flank of nude mice at day 0. Mice were injected daily intraperitoneally with 100 µl of 1 µmoles of orexin-A/Kg solutions starting at day 0 for both cell lines (○) or day 28 for OX1R/HPAF-II cells (▲) or with 100 µl of PBS (●) for controls. The development of tumors was followed by caliper measurement; (**C**) Formalin-fixed xenografted HPAF-II or OX1R/HPAF-II tumors from nude mice intraperitoneally injected daily or not with 1 µmoles/Kg orexin-A were analyzed by cleaved caspase-3 immunostaining. Cleaved caspase-3 positive cells were counted in 10 different fields, each comprising 500 tumoral cells, in the presence (black bars) or absence (white bars) of 50 days orexin-A treatment. Data are the means ± SE of 6 tumors in each group; $^{***}p < 0.01$ versus control.
